# Targeting *agr*- and *agr*-Like Quorum Sensing Systems for Development of Common Therapeutics to Treat Multiple Gram-Positive Bacterial Infections

**DOI:** 10.3390/s130405130

**Published:** 2013-04-18

**Authors:** Brian Gray, Pamela Hall, Hattie Gresham

**Affiliations:** 1 Department of Pharmaceutical Sciences, College of Pharmacy/MRF 208, MSC09 5360, University of New Mexico, Albuquerque, NM 87131-0001, USA; E-Mail: phall@salud.unm.edu; 2 Research Service, New Mexico Veterans Affairs Medical Center, Albuquerque, NM 87108, USA; 3 Department of Internal Medicine, Division of Infectious Diseases, University of New Mexico, Albuquerque, NM 87131, USA; E-Mail: hgresham@salud.unm.edu

**Keywords:** quorum sensing, peptide pheromones, bacterial pathogenesis

## Abstract

Invasive infection by the Gram-positive pathogen *Staphylococcus aureus* is controlled by a four gene operon, *agr* that encodes a quorum sensing system for the regulation of virulence. While *agr* has been well studied in *S. aureus*, the contribution of *agr* homologues and analogues in other Gram-positive pathogens is just beginning to be understood. Intriguingly, other significant human pathogens, including *Clostridium perfringens*, *Listeria monocytogenes*, and *Enterococcus faecalis* contain *agr* or analogues linked to virulence. Moreover, other significant human Gram-positive pathogens use peptide based quorum sensing systems to establish or maintain infection. The potential for commonality in aspects of these signaling systems across different species raises the prospect of identifying therapeutics that could target multiple pathogens. Here, we review the status of research into these *agr* homologues, analogues, and other peptide based quorum sensing systems in Gram-positive pathogens as well as the potential for identifying common pathways and signaling mechanisms for therapeutic discovery.

## 1. Introduction to *Staphylococcus aureus* and *agr*

### 1.1. Introduction

The contribution of communication systems within human bacterial pathogens to gene regulation has significantly altered our comprehension of how pathogens adapt to specific niches to promote disease. Cell-to-cell communication mediated by quorum sensing (QS) within a single species coordinates cooperative behavior to enhance survival under stress, alter metabolism, and savage host tissues and immune defenses. The accessory gene regulator (*agr*) operon-encoded QS system in *Staphylococcus aureus* is one of the most well studied communication schemes of human bacterial pathogens and numerous reports have demonstrated that QS is critical to the pathogenic abilities of this Gram-positive (G+) bacterium. The sensing of, and response to, the *agr*-encoded auto-inducing peptide pheromone (AIP) rapidly changes the expression of hundreds of genes to promote invasive infection and virulence in host tissues [1,2]. In fact, transcriptional analyses of isolates from skin and bone abscesses clearly reveal an important role for *agr* in acute human infections [3]. In contrast, *agr* dysfunctional isolates are associated with chronic infections and represent a minority of clinical isolates [4]. While these isolates are capable of colonization [5] and nasal carriage is associated with the development of these infections and is postulated to be their source [6], *agr* dysfunctional isolates do not persist in natural populations, indicating that *agr* mutants do not contribute to transmission where *S. aureus* infections are endemic [7]. Additionally, when *agr*-deletion (Δ*agr*) strains are tested in various infection and pathogenesis models *in vivo*, the bacteria may colonize but disease is attenuated [8–12], and clearance of individual *S. aureus* cells by host defenses is enhanced [9,13].

We and others are actively investigating host defense mechanisms that interfere with *S. aureus agr*-mediated communication with the goal of identifying therapeutic targets that limit disease and control infection without engendering resistance due to selective growth pressure [14–17]. Importantly, recent studies employing both traditional methods and bioinformatics techniques have revealed that G+ bacterial pathogens across the phylum of Firmicutes encode and express either homologues and analogues of the *agr* operon or similar QS systems that use small peptide “quormones” to regulate pathogenesis [18–24] (Table 1, see [25] for a description of the Quorumpeps database, available at http://quorumpeps.ugent.be, which provides multiple tools for investigating peptide quormones). Together these observations hint at the potential for development of anti-virulence compounds that are efficacious in numerous G+ pathogens. Whereas a single compound has been reported to inhibit common communication systems equally across multiple Gram-negative (G-) pathogens with therapeutic benefit [26], an anti-QS compound efficacious *in vivo* for multiple G+ pathogens has not yet been described. Anti-virulence strategies employing either drugs or vaccines could be significant adjuncts to the use of antibiotics in the treatment of infectious diseases [14,15,27,28]. Therapeutics that target virulence could aid antibiotic stewardship by limiting exposure of pathogens to antibiotics that drive resistance. To accomplish this goal, they could replace the use of prophylactic antibiotics sparing both exposure of the pathogen to antibiotic selection and disruption of the host microbiota. In addition, they could be used in lieu of antibiotics in clinical situations like uncomplicated skin and soft tissue infections in normal adults where host systems are sufficient to clear infection following surgical incision and drainage [29].

Moreover, they could aid existing antibiotics by facilitating host-dependent clearance of the pathogen rendered avirulent by the drug or antibody. Intriguingly, the development of a compound that works for multiple pathogens would increase the clinical utility of this approach. While the development of resistance to QS inhibitors has been postulated, recent studies in the G- pathogen *Pseudomonas aeruginosa* suggest that QS-insensitive mutants, which would be resistant to QS inhibiting anti-virulence therapies, form self-limiting populations in diseases where QS is required for pathology and dissemination [69,70] Thus, QS inhibition in G+ pathogens could be therapeutically beneficial without contributing to the spread of QS mutants. Here, we review what is currently known about the similarities in structure and function of *agr*, *agr*-like systems, and other peptide based quorum sensing systems across several human pathogens with the intent to highlight possible molecular targets for chemotherapeutic intervention against G+ bacterial quorum sensing.

### 1.2. Structure and Function of the agr Operon and AIP

**Figure 1.**
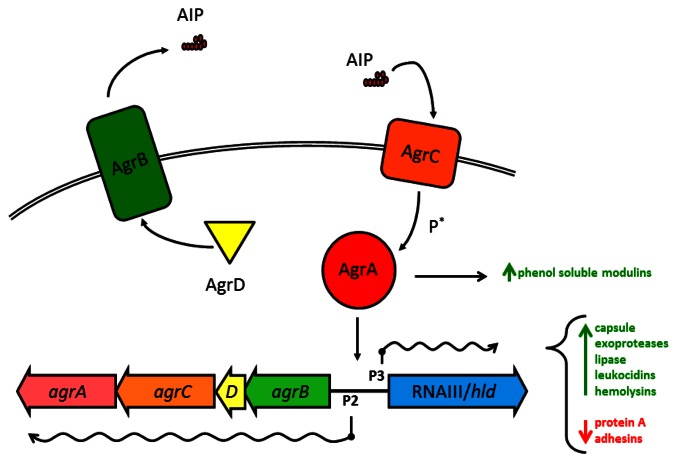
The structure and function of the *agr* operon in *S. aureus*. AgrB is a multifunctional endopeptidase and chaperone protein, and it has been suggested that AgrB is also involved in the export of AIP. AgrD is a propeptide processed by AgrB into the small thiolactone AIP. AgrC is the integral membrane sensor part of a two-component regulatory system. AgrA is the transcription factor response regulator companion to AgrC, and acts on the divergent P2/P3 promoter to upregulate *agr* and RNAIII expression, in addition to several other transcriptional targets. The regulatory RNA molecule RNAIII acts on numerous gene transcripts to modulate gene expression through post-transcriptional control.

**Figure 2.**
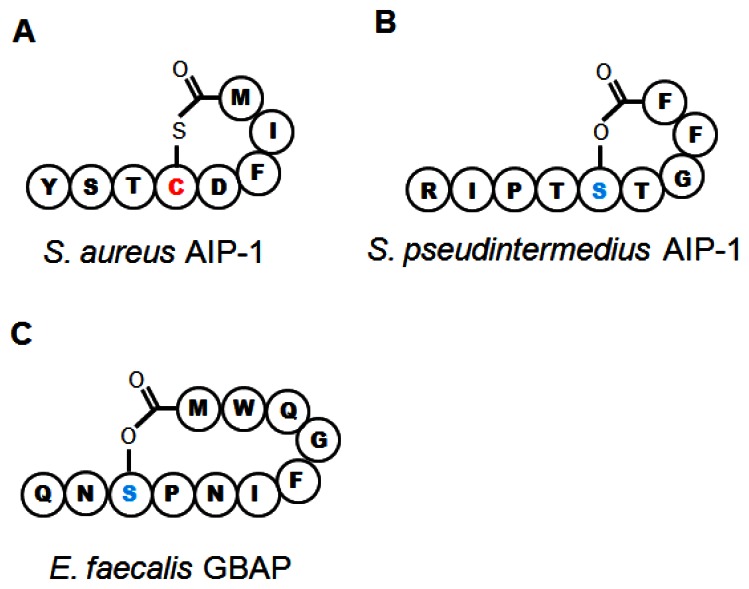
Structures of thiolactone and lactone signal peptides. (**a**) Structure of the prototypical autoinducing peptide, *S. aureus* AIP-1. (**b**) AIP-1 from *S. pseudintermedius*, the only reported *Staphylococcus* species with a lactone autoinducing molecule. (**c**) Gelatinase biosynthesis activating peptide (GBAP) from *Enterococcus faecalis*.

Substantial work has gone into understanding how the various Agr components within *S. aureus* interact (see Novick and Geisinger 2008 [71], and Thoendel *et al.* 2011 [2] for in-depth reviews). Briefly, the four genes in the *agr* operon are read as a single polycistronic message in the transcriptional order of *agrBDCA* (see Figure 1). AgrD is a short polypeptide which includes the protein sequence for AIP, but AgrD undergoes significant processing before the signal peptide is released. In the model proposed by Thoendel *et al.* [2], AgrD associates with the inner leaflet of the plasma membrane where it serves as the ligand for AgrB [72]. The cytoplasmic face of AgrB has several functions, including a sequence-specific protease that likely recognizes conserved residues that flank the central AIP sequence in both directions. AgrB cleaves the C′ terminus of AgrD and then catalyzes the formation of the thiolactone ring that defines the AIP structure (Table 1, Figure 2(a)) [73]. AgrB has been suggested to aid in translocating the partially processed AgrD to the outer leaflet of the plasma membrane, but this remains unclear [2,74]. The type I signal peptidase SpsB completes the N′ terminal cleavage of AgrD, releasing fully formed AIP from the cell surface [75].

The receptor for AIP, AgrC, is an integral membrane protein, and a member of the class 10 receptor histidine protein kinases (HPKs) with homology to members of the LytST/R two-component regulatory system (2CRS) family. AgrC has a high affinity for AIP, with activation EC_50_ values in the low nanomolar range. This exquisite sensitivity may serve as a defense mechanism for *S. aureus*, as we have previously shown that a single cell enclosed in a small space, such as the phagosome of a macrophage, can secrete sufficient AIP within a short time to trigger the *agr*-mediated QS transcriptional program [76]. AgrC can dimerize without binding AIP and binding even a single AIP molecule is sufficient to activate the receptor complex [77]. The two cytoplasmic HPK tails of AgrC cross-phosphorylate to allow AgrC to in turn activate the response regulator module AgrA.

Like AgrC, the transcription factor AgrA shares significant homology with LytST/R family members, and the consensus binding sequence has been identified for AgrA, with unsurprising similarities to the sequence for LytTR binding [78]. The best known target for AgrA binding, and the region most important for virulence regulation in *S. aureus*, is the divergent promoter region P2/P3 which controls transcription of the *agr* operon and the RNAIII/*hld* gene, respectively. Due to a two-nucleotide substitution in the consensus binding sequence in the P3 promoter, phospho-AgrA displays a preference for P2 at low concentrations, promoting an autoactivation circuit for *agr* [78]. Such positive feedback loops have been identified in other examples of QS in numerous species, and these circuits foster accelerated accumulation of the QS pheromone and rapid activation of the target transcriptional program. At high concentrations, active AgrA may slightly prefer P3 over P2 [79], but both *agr* and RNAIII are actively transcribed.

The sequence of the *agr* operon is not the same in all *S. aureus* strains. Four common variants, designated *agr* I through *agr* IV (Table 1), have been identified in clinical isolates and these are individually associated with various clusters of *S. aureus* isolates according to pulsed-field gel electrophoresis (PFGE) type; for example, the most common CA-MRSA isolate worldwide, PFGE type USA300, is an *agr* I strain (Figure 2(a)), while the PFGE type USA400 strain MW2 is *agr* type III. These types have arisen due to the presence of a hypervariable region in the *agr* operon, which encompasses the 3′ region of *agrB*, all of *agrD*, and part of the 5′ region of *agrC* [43]. Recent work from Geisinger *et al.* established that the four *agr* genotypes function with different kinetics, in that each *agr* allele activates gene regulation at distinct times during log phase growth and by varying degrees [80].

The AIPs encoded by the four *agr* types have slightly different lengths and peptide sequences (Table 1). The conserved central cysteine residue required for formation of the thiolactone bond is followed by four more amino acids to the C′ terminus (Figure 2(a)). The length of the N-terminal “tail” varies and all known AIP variants in *S. aureus* and other species range from 7 to 9 residues *in toto*. Of the four AIP molecules, AIP 1 and AIP 4 differ by a single amino acid at position 5 (Table 1) and have some cross-reactivity for their cognate AgrC receptors. However, the differences in peptide sequence between AIP 1/4, 2 and 3 allows these molecules to function as cross-type antagonists for AgrC activation, with low nanomolar IC_50_ values, apparently granting competitive advantages to competing strains of *S. aureus* [30,81,82]. The therapeutic potential inherent in these natural peptide analogs will be discussed below.

### 1.3. Gene Regulation by agr and RNAIII

The *agr*/RNAIII virulence regulon is one of the largest and most complex prokaryotic transcriptional programs currently known and reports continue to emerge of new virulence factors expressed under the *agr* regulatory scheme. Broadly, activation of *agr*/RNAIII triggers the transition from production of surface-bound proteins to the secretion of soluble exotoxins and degradative enzymes. Much of this complexity stems from the chromosomal juxtaposition of the *agr* QS system and the RNAIII molecule, combining the regulatory network of a 2CRS with a pleiotropic regulatory RNA (Figure 1, [1,83]). Appropriate to such an energetically taxing transcriptional program, upstream regulation of the *agr* operon is temporally, nutritionally, and spatially complex.

The two key transcription factors that positively regulate expression of the *agr* operon are staphylococcal accessory regulator A (SarA) and AgrA itself [84–86]. Deletion of *sarA* dramatically alters both the level and timing of RNAIII production in response to AIP signaling by markedly reducing transcription of the *agr* operon and deletion of *agrA* completely abolishes both RNAIII and *agr* mRNA [86]. This finding demonstrates that AgrA binds the P2 sequence at constitutively low levels even in the absence of an active AIP-driven signal through AgrC and that AgrA may be the most important element in the initiation of transcription at P2. MgrA, a transcription factor involved in control of multidrug resistance and cell wall turnover, along with other *sar* family members SarU and SarZ, all contribute positively to *agr* expression [87–89]. MgrA also promotes expression of SarX, a repressor of *agr*, giving *S. aureus* an “off-switch” for the *agr* signaling cascade [88]. The metabolic sensor and transcriptional repressor CodY prevents *agr* transcription under conditions of enriched nutrition; once key nutrients are depleted below a threshold, CodY can no longer bind to the P2/P3 region, derepressing *agr* [90,91]. σ^B^, an alternative transcription factor produced as part of the broad stress response in *S. aureus*, strongly promotes biofilm production and is an additional inhibitor of *agr*, although its mechanism of action remains unclear [2,92,93]. In addition, redox signaling controls AgrA-DNA interactions via oxidation of a critical cysteine residue within AgrA that terminates its transcriptional activity [94].

For all of the regulatory checks and balances upstream of *agr*, AgrA as a transcription factor directly regulates only a few promoter sequences in addition to P2/P3 (Figure 1), notably the α- and β-phenol soluble modulins (PSMs) [1]. PSMs are alpha-helical, amphipathic cytolytic peptides that range in size from ∼20 to 50 aa. They are genome encoded and most strains produce PSMs. *S. aureus* produces 4 PSMα peptides encoded in the *psma* operon that are homologous to the RNAIII-encoded δ-toxin and two PSMβ peptides encoded in the *psmb* operon [9]. This RNAIII-independent but *agr*-dependent regulation of these multifunctional cytolytic peptides suggests that QS regulation of PSMs evolved prior to the addition of a wider control of virulence to the *agr* regulon [1]. This may well have occurred by expansion of the RNAIII-encoding region around the initial gene for δ-toxin. While these data have been confirmed primarily in the strain MW2, they suggest that the RNAIII-independent but *agr*-dependent suppression of carbohydrate metabolism confirmed by microarray may reflect a wider contribution of *agr*, possibly through AgrA, to metabolism [1].

RNAIII is one of the largest known prokaryotic regulatory RNA (rRNA) molecules and facilitates the greater part of *agr*-dependent virulence via interactions mediated by specific regions of RNAIII secondary structure (for a recent review see Felden *et al.* [83]). The only protein translated from RNAIII is the small peptide δ-toxin, as the *hld* gene is encoded within the first half of the sequence of RNAIII, but the protein is not produced until significantly after RNAIII has been transcribed [95]. For some genes, like α-hemolysin, RNAIII binding derepresses translation initiation by opening the ribosomal binding site in the target mRNA. Various structures within RNAIII also work via antisense RNA mechanisms to prevent translation initiation in target mRNAs which often accelerates mRNA turnover by RNAse digestion of extended dsRNA structures. Many of the target genes shut down by RNAIII through post-transcriptional mechanisms are key members of other regulatory pathways, like repressor of toxins (*rot*), members of the Sar and MarR families of transcriptional regulators, and multiple 2CRS modules that can variously promote and repress expression of hundreds of toxins, proteases, adhesion factors and metabolic pathways [1,2,83,96]. RNAIII also directly affects the transcripts of many genes whose expression is regulated by Rot and other transcription factors, so that total cellular mRNA turnover increases dramatically after AIP signaling. This combination of direct and indirect regulation by RNAIII rapidly transforms *S. aureus* cells from a sessile, stationary state into an aggressive planktonic form capable of invasive infection.

Microarray analyses of the *agr*-regulated transcriptome in various *S. aureus* strains have identified core virulence factors that depend either on RNAIII or AgrA for regulation [1,31,32]. These include genes for PSMs, capsule production, α-hemolysin, cytolytic toxins, proteases and lipases. However, there is considerable variability in the total number of genes regulated as well as their contribution to genetic programs that regulate growth and survival. This fact suggests that inclusion of genes into the *agr*-regulated transcriptome could represent an adaptive response to alter not only virulence factors but also metabolic pathways required for survival within distinct niches.

### 1.4. agr in *S. aureus* Disease

*Agr* involvement in disease is complex, and numerous groups have worked to clarify the role of *agr* in *S. aureus* disease, but varying infection and disease models, and variable patient populations, cloud the issue. Nevertheless, it is becoming clear that *agr* plays an important role in promoting acute and aggressive infection in both animals and humans. For example, murine dermonecrosis is an acute model of invasive skin and skin structure infection characterized by development of a subcutaneous abscess and a superficial ulcer after subdermal injection of *S. aureus*. Deletion of *agr* blocks ulcer formation and reduces abscess size [10], a result also observed when PSMαs are deleted [9] or α-hemolysin is blocked by vaccination [97]; both gene products are tightly regulated by *agr* in many *S. aureus* strains. Of all the *agr*-regulated toxins, the PSMs are among the most important in enhancing the survival and dissemination of *S. aureus* in invasive infection. PSMαs are cytolytic against neutrophils, although this enhances expression of inflammatory molecules in mouse models of dermonecrosis [9] *Agr* is also required for development of invasive, necrotizing pneumonia in mice [10,98,99], although the inflammatory response in the lung does not require *agr* function [98]. The pathology of *S. aureus* osteomyelitis in rabbits is reduced in the absence of *agr* [11], and the early colonization of heart valves is markedly reduced in rabbits intravenously injected with a Δ*agr* strain [100].

These findings derive from acute infection models, with readouts in hours or days after infection, and they contrast with the literature about chronic *S. aureus* infections in humans. Patients suffering from persistent *S. aureus* bacteremia are more likely to be carrying *agr*-deficient strains, a pool of point mutations which is also associated with a higher incidence of chronic infective endocarditis [101]. Similar studies confirmed that *S. aureus* pools from chronically infected patients accumulate mutations rendering *agr* inoperative, and these isolates are associated with increased hospitalization rates [5,7,102]. Another group reported that loss of expression of Panton-Valentine leukocidin and increased expression of protein A, a phenotype correlating to a loss of *agr* function, was associated with well-established, active *S. aureus* infection [3]. A recent report by Gagnaire *et al.* found a strong association between the duration of chronic infections, the loss of *agr* function, and an increase in glycopeptide resistance [4]. Overall, *agr* deficiency is more commonly found in hospital-acquired methicillin resistant *S. aureus* (HA-MRSA) than in community acquired MRSA (CA-MRSA) isolates [5,102] which may be due in part to the finding that expression of methicillin-resistance gene *mecA*, commonly found in HA-MRSA isolates, inhibits *agr* function [103].

Aggressive CA-MRSA infections have been hypothesized to require *agr* expression to maximize host-to-host transmission and early colonization [7,8], but that HA-MRSA infections may not rely on *agr* for transmission because surgical and other medical procedures can bypass human infection barriers such as the skin and mucus membranes. Alternatively, constant activation of the *agr* regulon is suggested to be energetically expensive. Therefore, transition to a metabolically favorable *agr*-negative status is beneficial for long-term survival once innate host defenses have been eluded [8,104,105].

One noted phenotype associated with the loss of *agr* is the development of thicker biofilms [93,106–108], a behavior that likely stems from *agr* regulation of PSMβ and/or protease production. PSMβs are crucial to biofilm maturation and dissemination in *S. aureus* and foci of *agr*-mediated PSM promoter activation can be observed within static biofilm structures [109]. Single *S. aureus* cells can produce sufficient AIP to activate quorum sensing within confined spaces [76], and this ability may explain how *agr*-mediated QS controls *S. aureus* dissemination from within mature biofilms. Thicker, *agr*-deficient biofilms with limited dissemination may serve as incubators for the development of antibiotic resistant strains [110,111]. In cases of *S. aureus* biofilm formation on medical implants and indwelling lines, loss of *agr* could prove somewhat beneficial as this would inhibit biofilm maturation and retard bacterial dissemination [106,109], thus reducing the spread of infection.

### 1.5. Host and Molecular Antagonism of S. aureus agr

Mounting evidence suggests that the story of *S. aureus* and humans is one of co-evolution and co-adaptation [112,113]. While *S. aureus* may thrive in the anterior nares as a commensal, colonization elsewhere in the body is met with vigorous immune response, as demonstrated by the development of defenses that target many of the effectors of *S. aureus* pathogenesis. Our adaptive immune system works to thwart chronic or recurrent infections, often by producing antibodies against capsular polysaccharides or exotoxins. Ongoing efforts to develop suitable vaccine epitopes derived from many virulence factors have met with mixed success [97,114–116]; their failures to protect against a broad assortment of *S. aureus* strains are likely due to the marked genetic variability and pathogenic profiles between isolates. As a small peptide, AIP is not particularly immunogenic and thus the host adaptive immune system does not regulate QS. To address whether adaptive immunity could provide protection by targeting QS, Park *et al.* demonstrated that a synthetic analog of AIP-4, when linked to a strongly immunogenic hapten, could be used to develop an *agr*-blocking antibody in mice. This antibody prevented tissue damage in a mouse model of dermonecrosis when it was passively administered at the time of infection with the AIP 4 strain RN4850 [117]. This finding confirmed that specific targeting of *agr*-mediated QS is a valid therapeutic approach to limit pathogenesis, although it requires development of a multi-type vaccine to be broadly effective against *agr*-mediated diseases.

We have other innate immune defenses that work to quench or disable the *agr*-driven signaling cascade. While low pH is an effective physical barrier and is also used by phagocytes to digest many bacteria, *S. aureus* is notoriously acid tolerant, with an acid shock response that allows it to survive prolonged exposure to low pH [118]. However, it was observed 20 years ago that low environmental pH inhibits *agr*-mediated pathogenesis [119] due to the acid shock transcriptional program down-regulating *agr* expression [120]. Phagocytes, neutrophils and macrophages also produce reactive oxygen species like hypochlorite and superoxide which, in addition to damaging bacterial membranes and extracellular proteins, also oxidize and inactivate AIP-1 [121], the *agr* type most commonly found in *S. aureus* infections. In addition, there are two common components in blood which can quench AIP signaling: hemoglobin and apolipoprotein B (ApoB) [122,123]. ApoB found in low- and very-low-density lipoproteins (LDL/VLDL) binds AIP directly and sequesters it thus preventing ligand activation of AgrC [123]. The α- and β-chains of hemoglobin also antagonize *agr* signaling, most likely by inhibiting AIP secretion following hemoglobin-mediated membrane disruption [122,124,125]. These innate defense mechanisms are employed when inflammatory cytokines induce leaky vascular endothelium and serum components enter the site of infection.

Besides host mechanisms that counter *agr*-mediated QS, there are numerous examples of molecules produced by other strains of *S. aureus* and by other species of bacteria that interfere with *agr* signaling. As described above, different AIP types can antagonize non-cognate AgrC receptors to prevent activation of the *agr* regulon, even after only a single application of inhibitory AIP [2,30,126]. Many other *Staphylococcus* species are also *agr*^+^ (Section 2.1) and some research has suggested that *S. epidermidis* AIP molecules antagonize several of the *S. aureus* AIP genotypes *in vitro* [127,128], but *in vivo* experiments have not yet confirmed this finding [129]. Some bacterial species disrupt *agr*-mediated QS in *S. aureus* using molecules distinctly unlike AIP. Several reports demonstrate that members of the *Lactobacillus* genus produce anti-QS molecules against both G- and G+ organisms, and Li *et al.* recently reported on a dicyclic peptide from *L. reuteri* that quenches *agr*-mediated production of toxic shock syndrome toxin across multiple *agr* genotypes [130], although the mechanism of interference is currently unknown. *Pseudomonas aeruginosa* produces at least two molecules that interfere with *agr*-mediated QS in *S. aureus*; one is a long-chain acylhomoserine lactone (AHL) that *P. aeruginosa* employs for its own QS processes [131], and another is an oxidized quinoline [132]. AHL binds to a saturable but unknown receptor to inhibit AIP signaling, and the quinoline may function through destabilizing membrane proteins to interrupt cell functions. In light of the dawning appreciation that humans are host to polymicrobial communities across all of our diverse epithelia, these observations support the concept of probiotic treatments and therapies derived from inter-species bacterial warfare.

## 2. *agr* and Analogous Peptide Quormone Systems

### 2.1. agr Homologues

Elegant in its organization and compact in its function, *agr* is the prototype for small peptide quormone QS systems in G+ bacteria. As described previously, the *agr* operon encodes for the complete set of functions necessary for a density-dependent master regulator of bacterial programming. In *S. aureus*, *agr* activation effects a marked shift in the transcriptome and in the metabolic capability of responding cells, and this is reflected in the reported effects of *agr* signaling in those species with *agr* homologues, as described below. In comparison, non-*agr* peptide quormone systems do not trigger changes in species behavior as dramatic as those seen in *S. aureus*, even though these other systems play an important role in promoting virulence. Below we describe the role of *agr*-mediated QS processes in controlling virulence behaviors in several species.

#### 2.1.1. Staphylococcal *agr*

The *agr* operon is found throughout the genus of *Staphylococcus* in pathogenic, commensal, and environmental species with genetic variation in the operons closely following the 16S rRNA phylogenetic tree [24]. Although the function of *agr* has not been explored in all of these species, there are several notable staphylococcal pathogens besides *S. aureus* where expression of virulence factors is regulated by *agr*. Of these *Staphylococcus* species, *S. epidermidis* is currently recognized as the most clinically important nosocomial pathogen, due in part to its ubiquitous presence on human skin and mucus membranes [133]. Unlike *S. aureus*, *S. epidermidis* is rarely associated with invasive infection. Instead, *S. epidermidis* forms tenacious biofilms growing on in-dwelling lines and surgical implants, so that most infections are sub-acute or chronic. However, *agr* is important for *S. epidermidis* virulence [34,35], and there are at least three different AIP types for *S. epidermidis* (AIP-Se, Table 1). *Agr* signaling in *S. epidermidis* induces production of RNAIII/δ-toxin, exoproteases, a lipase, and PSMs [19,34,36], but also promotes the expression of numerous adhesins including polysaccharide intercellular adhesin PIA [33]. An *agr*-deleted strain was unable to produce PSMs and elicited less neutrophil migration and inflammatory cytokine production compared to wild-type *S. epidermidis* [36]. Many of the genes affected by AIP-Se signaling belong to metabolic pathways, suggesting that *agr*-mediated QS prepares *S. epidermidis* for the stationary, sessile lifestyle within a biofilm [19,38]. Intriguingly, in addition to increasing biofilm deposition *agr* regulation also enhances biofilm remodeling, maturation and dissemination through expression of PSMβs [134]. In contrast, Dai *et al.* recently reported that *S. epidermidis* isolates recovered from in-dwelling catheters produced thicker biofilms and exhibited more autolytic behavior in concert with down-regulation or deletion of the *agr* operon and upregulation of the autolysin *atlE* [135] suggesting that *agr* regulation of biofilm formation is carefully counterbalanced by other factors. From these reports it is clear that *agr* regulates pathogenesis in *S. epidermidis*, making the *agr* QS system an attractive target for pharmacotherapeutic control of *S. epidermidis* infection.

*S. lugdunensis* is a commensal skin organism but is also a highly virulent, opportunistic pathogen responsible for endocarditis, septicemia, osteomyelitis, and skin and soft tissue infections. Known virulence factors include δ-toxin, lipase and a polysaccharide capsule, and some reports suggest that different strains are positive for a membrane-bound coagulase or α-toxin [136,137]. *S. saprophyticus* is an important pathogen in urinary tract infections and it expresses numerous adhesins that bind to extracellular matrix proteins although it produces no known toxins. Both species produce AIP (Table 1) and their *agr*/RNAIII are organized similarly to those of *S. aureus* and *S. epidermidis*, but the *hld* gene for δ-toxin is not embedded within RNAIII in *S. lugdunensis* or *S. saprophyticus* [40,43,138]. At this time there are no animal models of pathogenesis for either species, so the relevance of *agr*-mediated QS to *in vivo* virulence regulation is unknown.

The *S. intermedius* group includes three species that, like *S. aureus*, straddle the commensal-pathogen divide in their respective hosts. The best studied of these, *S. pseudintermedius*, is a gastrointestinal commensal in dogs, but it is the major cause of canine pyoderma and can very rarely infect humans [139]. Along with recent taxonomic reclassification separating *S. pseudintermedius* from *S. intermedius* [42], a complete sequence of the *S. pseudintermedius* genome has recently been published, demonstrating that it carries genes for multiple toxins, many of which are highly homologous to similar toxins in *S. aureus*, including leukotoxins and α-, β-, and δhemolysins [140]. *S. pseudintermedius* AIP was first identified in 2003, and biochemical characterization determined that instead of forming a thiolactone like that found in AIP-Sa, the nonamer AIP-Si relied on a central serine residue to form a lactone ring (Table 1, Figure 2(b)) [141]. This molecule is functional for QS in *S. pseudintermedius*, and substitution of a cysteine for the key serine molecule reduced the expression of RNAIII [41]. Three other genetic variants of AIP have since been identified in *S. pseudintermedius* (Table 1, [41,42]), but whether *agr*-mediated QS controls pathogenesis in this species remains to be determined.

Genomic and proteomic analyses of other *Staphylococcus* species have demonstrated that numerous clinical and environmental isolates include an *agr* operon and likely produce their own version of AIP [18,30]. However, lacking medical or veterinary impact, the role of QS in modulating pathogenesis in these other species has not been examined.

#### 2.1.2. Clostridial *agr*

Species in the order Clostridiales generate some of the most potent bacterial toxins known, ranging from the severe neurotoxins of *C. botulinum* and *C. tetani*, to a range of aggressive proteases produced by *C. perfringens*. While the role of *agr* in virulence programming in some clostridial species has yet to be determined, it is evident that *agr* controls toxin production in *C. botulinum* and *C. perfringens*. Pathogenic *Clostridium* species are generally resistant to multiple antibiotics, thanks to the slow growth of most species, obligate anaerobic life style, and their ability to form endospores, a non-metabolizing and durable cell form highly resistant to heat, desiccation, radiation, oxidation and numerous other chemical means of microbial control. Therefore the role of QS in controlling toxin production and metabolic alteration in these species is receiving a great deal of attention and several recent reports have demonstrated that *agr*-mediated signaling regulates both toxigenesis and sporulation (Table 1).

Botulism intoxication in healthy adults is not normally due to infection with *C. botulinum*, although colonization of the gut is the accepted mechanism for infant botulism. In adults, rather, consuming preserved food initially contaminated with botulism spores leads to ingestion of the botulism neurotoxin, of which there are several types. Cooksley *et al.* demonstrated that Group 1 *C. botulinum* expresses two AgrBD homologues (Table 1) and the two signaling peptides play different roles in controlling pathogenesis [44]. mRNA expression of both *agrBD1* and *agrBD2* peaks late in exponential growth and markedly decreases in stationary phase growth, typical of genes involved in QS. But while *agrD1* expression regulates sporulation, *agrD2* controls toxin production during growth *in vitro*. The cognate sensors or 2CRS for these two *agrD* homologues have not yet been described.

*C. difficile* infection is associated with severe diarrhea due to clostridial overgrowth and tissue adhesion after the patient's normal gastrointestinal flora has been eliminated due to antibiotic therapy. Sequencing the complete *C. difficile* genome has revealed the presence of both a complete *agrBDCA* operon as well as a second copy of *agrBD* ([45,142], Table 1), but currently their roles in pathogenesis *in vivo* are unknown.

Infection by *C. perfringens* can also lead to gastrointestinal distress, but this species is also the causative agent for gas gangrene. Deep wounds can give rise to anaerobic conditions, and thus provide an ideal environment for infection by environmentally ubiquitous *C. perfringens* endospores. Germinating *C. perfringens* produces numerous proteases and toxins to digest host tissues, which in turn supply necessary amino acids and nutrients for which *C. perfringens* lacks the necessary biosynthetic pathways. This exotoxin-mediated release of nutrients *in situ* rapidly accelerates bacterial growth, further complicating the treatment of *C. perfringens* infection. Several reports have demonstrated that *agrBD* (Table 1) is important for toxin production and sporulation in many of the pathogenic groups of *C. perfringens*, including both human and veterinary isolates [21,46,47,143,144]. Epsilon-toxin and beta 2 toxin production by Δ*agrB* Type B strains was reduced compared to wild-type bacteria in *in vitro* assays using enterocyte-like CaCo2 cells [47,144], and *agrB* controls *in vivo* toxicity and colitis in a rabbit intestinal loop model with a Type C strain [143]. Remarkably, in at least one Group A strain *agrBD* regulates expression of genes on a plasmid [21], a novel result previously unheralded in the *agr* literature. The cognate receptor for *C. perfringens* AIP has not been conclusively identified, but in Type A strains the 2CRS *virRS*, which regulates expression of several toxins and virulence factors through the regulatory RNA molecule VR-RNA [145,146], may be involved in *agr*-mediated QS and pathogenesis [46]. However, the link between *agr* and *vir* is unclear in other strain types, as VirRS does not appear to be involved in *agrBD*-mediated toxin production in Type B or D strains of *C. perfringens* [47]. While more research into the role of QS in *C. difficile* pathogenesis is required, it is clear that developing treatments to inhibit *agr*-controlled pathogenesis in *C. perfringens* and *C. botulinum* could prove beneficial to reducing disease severity.

#### 2.1.3. Enterococcus

*E. faecalis* resides in the human gastrointestinal tract as a member of the endogenous flora, but it can persist in the face of numerous environmental hazards, and vancomycin-resistant strains are nosocomial pathogens of increasing importance. *E. faecalis* infections of the urinary and seminal tracts include urethritis, prostatitis and nephritis, and it can also invade the kidneys, giving rise to bacteremia and endocarditis. Virulence factors, several of which are regulated by QS processes, include numerous adhesins and biofilm components but the best characterized are proteases, gelatinase (GelE) and a serine protease (SprE), that enable *E. faecalis* to invade host tissues. In particular, gelatinase, a metalloprotease, aids in digesting host extracellular matrix proteins and also promotes biofilm dissemination [147,148]. The *fsr* operon regulates QS-mediated pathogenesis through control of GelE and SprE expression, down-regulation of biofilm components, and alterations in metabolic function [149–151] (Table 1). FsrD, the propeptide for the GBAP quormone, (Table 1) is cleaved, cyclized and exported by FsrB [50,152]. *Fsr* organization differs from *agr* as *fsrD* is contained entirely within the sequence of *fsrB* in a different frame, and is read from a distinct transcriptional start site [48]. Furthermore, GBAP forms a lactone ring four residues larger than the prototypical AIP thiolactone (Figure 2(c)). However, FsrA and C are highly homologous to AgrA and C, respectively, and there are two genes adjacent to *fsr* that are FsrA targets [50,152], echoing the operon and regulon structure of *agr*. Deletion or disruption of *fsr* appears to trigger a stress-response phenotype [153], enhances host survival in a mouse model of infectious peritonitis [50] and reduces tissue damage in a rabbit model of experimental endopthalmitis, suggesting that disruption of *fsr* regulation could have therapeutic benefits [148].

#### 2.1.4. Listeria

*Listeria monocytogenes* is the etiologic agent of listeriosis, a food-borne infection that causes a wide range of severe illnesses in the elderly, neonates and immunocompromised patients, and the *agr* operon assists this pathogen in persisting through harsh environments. *L. monocytogenes* forms biofilms on a broad variety of industrial abiotic surfaces including stainless steel and plastic, and these biofilms provide reservoirs for colonization of raw or partially-processed foodstuffs. Virulence factors include the toxins listeriolysin O (LLO), hemolysin, phospholipase, and several cell-surface proteins involved in promoting phagocyte internalization and actin polymerization. *L. monocytogenes* evades host immune defenses by breaching the vesicle membrane of a phagocytosing cell, where ActA-catalyzed formation of actin “tails” propels the bacteria through the cytosol and into a neighboring cell.

The *L. monocytogenes* genome encodes for a complete *agr* operon (Table 1), which plays a critical role in promoting biofilm production, expression of adhesion factors, and internalins, but does not affect growth (Table 1 [52,53]). *Listeria* lacks RNAIII, so the *agr*-mediated QS transcriptional program is controlled by direct AgrA binding of promoter sequences, and there is evidence that several dozen genes are both positively and negatively regulated [52]. A role for *agr*-mediated QS in virulence is less clear and may depend upon the growth phase of the inoculum administered. Mouse tail vein injections of a Δ*agrA* mutant grown to mid-exponential phase had attenuated virulence compared to the parental strain [51], whereas infection with a similar mutant grown to stationary phase demonstrated that loss of AgrA had no effect upon systemic virulence [154]. In contrast, *in vitro* cell invasion assays demonstrated that *agr* plays no role in maintenance of the intracellular life cycle that characterizes *L. monocytogenes* disease [51,154]. Many *L. monocytogenes* isolates are *agr*^+^ [23] suggesting that this locus plays an important role somewhere in the *Listeria* life cycle even if it is not essential for human infection, and that application of anti-*agr* therapeutics in industrial settings associated with food processing could limit the spread of listeriosis.

#### 2.1.5. Lactobacillus

No *Lactobacillus* species are yet identified as pathogens of either humans or animals, but they are of great interest in both food production and the burgeoning field of probiotic therapy. This approach aims to prevent pathogenic infections by prophylactically colonizing tissues with transient or potentially commensal bacteria with antimicrobial properties. Numerous studies have investigated the ability of sundry *Lactobacillus* species and similar lactic acid bacteria to generate anti-bacterial peptides such as bacteriocins, and other compounds like reuterin and nisin [155–157]. Among these, at least one species, *L. plantarum*, encodes multiple *agr* homologues [158]. The *lam* operon, structured as *lamBDCA*, is an *agr* homologue that produces a thiolactone peptide (Table 1) and is involved in bacterial adhesion to abiotic surfaces [54]. QS-mediated control of bacteriocin production in *Lactobacillus* species is regulated by other analogous systems, which will be discussed below.

### 2.2. Other Peptide Quormone Systems

While a few G+ pathogens are reported to employ AHL-based QS systems to regulate growth and toxin expression, the rest of the pathogens in the Firmicutes with QS-mediated control of virulence rely on small peptides as the quormone molecules. Outside of the *agr* family of homologues described above, there are primarily two other families of signaling systems found to regulate virulence. The RNPP family, best studied in *Bacillus* species and in *Enterococcus faecalis*, controls the production of numerous toxins and extracellular virulence factors [159]. The *com* family of parallel QS systems found in *Streptococcus* species triggers significant changes in bacterial lifestyle and behavior that can significantly enhance or promote virulence. Although components in individual examples of each of these systems may lack homology to members of the *agr* family, there are striking functional and behavioral similarities across these three different systems that suggest targeting QS-mediated control of virulence in these species could prove fruitful in inhibiting disease *in vivo*.

#### 2.2.1. *Bacillus* Systems

*Bacillus anthracis* produces a tri-partite cytotoxin and an anti-phagocytic capsule, and several strains of *B. cereus* are toxigenic and can cause brief but severe gastrointestinal distress. Research into the role of QS in virulence regulation in these pathogens is in its initial stages, but the action of peptide quormones in these species is not as clear-cut as with the *agr* operons. *LuxS*, a QS system best characterized in G- bacteria that uses AHLs as signaling molecules, plays a role in bacterial growth, and this system affects the expression of virulence factors in *B. anthracis* but its loss does not affect virulence [160–162]. In addition to the *luxS* system, numerous members of the *Bacillus* genus express members of the RNPP (Rap, NprR, PlcR, and PrgX) family of quorum sensing operons [56,60,163]. RNPP and *agr* share several crucial features, as both QS systems encode for a propeptide that is cleaved and secreted, and in the case of many RNPP members, the quormone is a heptapeptide [61,164]. Additionally, the cognate receptor has strong specificity for the co-evolved peptide [22,55]. But the two systems differ in that the RNPP peptides are linear, the processing enzyme(s) for the propeptide are not encoded in the same operon [165], and the signaling peptides must be re-imported into the cell via the Opp transporter before binding the cytoplasmic receptor [55,61].

In *B. cereus*, RNPP members regulate growth, sporulation, and toxin production (Table 1, [22,55,56]). The RNPP families regulate virulence in *B. cereus*, as it has been shown that a PlcR mutant strain caused less retinal epithelial cell death compared to wild-type bacteria in an *in vitro* model of endopthalmitis [57]. As shown in Table 1, there are five known *B. cereus* PapR signal peptide “pherotypes” analogous to the four *agr* types in *S. aureus*, and one report demonstrated that four of these pherotypes can cross-inhibit each other [164]. The fifth peptide, PapRa, was recently reported by Huillet *et al.* and plays a role in the oxidative stress response and sulfur metabolism during stationary phase [58] but its role in virulence, and its ability to interfere with other Pap quormones are yet to be determined. In *B. thuringinensis*, which is not pathogenic to humans but produces an insecticidal toxin, deletion of *papR*, which encodes the signal propeptide, reduced toxin production and the killing of susceptible insects [55]. Of note, NprR expression in *B. thuringinensis* is required for vegetative bacterial growth, biofilm formation and sporulation in insect cadavers [59], a novel finding demonstrating a role for quorum sensing behavior in post-pathogenic behavior. In light of this extant family of virulence-regulating QS systems, it remains to be determined whether an *agr*-like of QS system operon in *Bacillus* species operates in parallel to the RNPP family in regulating bacterial pathogenesis. Together, these studies suggest that at least 3 pathways in 2 QS systems contribute to pathogenic gene regulation in *Bacillus* species with significant potential for exploiting common strategies for modulation of biologic function.

#### 2.2.2. Streptococcal Systems

*Streptococcus pyogenes*, also known as Group A β-hemolytic streptococcus (GAS), is a commonly encountered G+ pathogen, and it gives rise to suppurative infections that range from mild (pharyngitis) to severe (necrotizing fasciitis). Different isolates of GAS display distinct preferences for either the skin or the throat as their site of infection, and this bias may be affected by the particular peptide quormone QS system encoded by a given isolate. GAS produce a wide range of toxins and virulence factors, including streptolysins, superantigens, a polysaccharide capsule, and proteases, and production of several of these virulence factors is affected by peptide quormone signaling.

The streptococcal *com* system, historically the first QS-mediated behavioral program described in the literature [166], controls bacterial competence in numerous α-hemolytic streptocci. It consists of two *com* operons; the first encoding a protease and dedicated peptide export channel and the second including a propeptide and a 2CRS sensor and regulatory modules [64,167]. ComC is cleaved to release the C′ terminal competence stimulating peptide (CSP), which is a 16-mer linear peptide (Table 1). Other α-hemolytic streptococci may also carry *com* with species-specific variations [167], but it has been suggested that several *S. pyogenes* isolates may have evolved without the ability to detect CSP in order to limit genetic variability [64]. In *S. mutans*, CSP signaling also regulates the production of biofilms and bacteriocins, enhancing both pathogenicity and competitive survival in the oral cavity [168–170]. *ComRS* constitutes a second QS-regulated competence system in *S. pyogenes* and in non-pyogenic streptococci that promotes competence in stationary phase cultures [67,171]. After export from the cytosol, the short polypeptide ComS is cleaved into XIP (Table 1) and then imported into the cell where XIP binds ComR. This protein is part of the Rgg family of transcription factors activated by binding small signal peptides. Once bound to XIP, ComR in turn promotes transcription of both ComS and SigX, an alternative sigma factor that promotes competence. The XIP quormone promotes competence in chemically defined media, but in more nutritionally complex environments, XIP signaling may promote bacterial cell death [172], possibly enhancing biofilm structure and mass through release of structural components such as extracellular DNA.

While competence is not directly related to bacterial pathogenesis in the majority of α-hemolytic streptococci, the ability to utilize exogenous genetic material clearly enhances the ability of streptococcal species to survive in a wide range of environments and under stressful conditions. This concept is supported by the finding that non-opsonizing antibodies against *S. pneumoniae* polysaccharide capsular antigens enhance transformation efficiency in pneumococci [173], potentially increasing bacterial pathogenesis and improving bacterial viability. However, currently there is no evidence the *com* operon or streptococcal competence play direct roles in GAS pathogenesis either *in vitro* or *in vivo*.

Genes in the streptococcal *fas* operon share homology with the 2CRS components of *agr*, as FasA shares sequence homology with AgrA, and FasB and C are strongly related to AgrC, although the ligand has not yet been identified for FasB or C [63]. Significantly, activated FasA promotes transcription of FasX, a small, regulatory RNA that binds the 5′ untranslated region of the streptokinase A (SKA) mRNA to increase transcript stability and message half-life [65]. SKA is a well-characterized virulence factor for skin-trophic GAS isolates that converts human plasminogen into the fibrin-cleaving protein plasmin. FasX, or possibly FasA, also appears to increase bacterial adhesion to human epithelial cells, promote internalization, and enhances host cell apoptosis and infection-mediated expression of the pro-inflammatory cytokine IL-8 [174]. Whether *fas* is involved in QS-mediated transcriptional programming is currently unclear, as the environmental/nutritional sensor CodY positively regulates FasA production [175]. This link suggests that early steps in control of *S. pyogenes* pathogenesis may be regulated more by locale within the host rather than bacterial density. This observation is in contrast to the finding that CodY represses *agr* function in *S. aureus* [90,91].

The streptococcal invasion locus (*sil*) uses a peptide pheromone and a 2CRS to limit the ability of Group A and Group G streptococci to invade host tissues while it enhances *Streptococcus* survival in the host. *Sil* is characterized by three transcripts covering two operons and a standalone gene. The locus sits between two of its major regulatory targets, the *blpM* and *blpU* operons that encode bacteriocin-like peptides [62,176]. The first operon, *silAB*, comprises the regulatory and sensor-HPK modules of the 2CRS. The second, *silEDCR*, includes two parts of an ATP-binding cassette transporter (SilD and E) and SilCR is a propeptide homologous to ComC that is processed into a 17-residue linear pheromone peptide (Table 1). SilCR signals through SilB to activate SilA, which increases transcription of *silEDCR*, *blpM* and *blpU* [62,177]. SilA also promotes expression of the virulence factors streptolysin S, iron transporter SiaA, and serine protease ScpC in a growth-phase dependent fashion [178]. The third *sil* mRNA is *silC*, which overlaps much of *silCR* and is transcribed in the opposite direction [62,176,177], but expression of *silC* is blocked in response to SilCR signaling and *silEDCR* transcription [177]. *SilC* contains a *com*-box [176], so it should be activated in the late exponential growth phase after *com*-mediated QS occurs and may promote systemic invasion of the host as part of a regulatory circuit with *silCR* [177]. In mouse models of necrotizing fasciitis, SilCR signaling appears to reduce lesion size and attenuate acute lethality due to systemic infection [176,179] but retards host healing of the initial lesion due to decreased clearance of GAS [178]. SilCR is able to simultaneously signal multiple isolates of both GAS and GGS [62] and there may be another, non-SilB receptor for the autoinducing peptide [176]. These data suggest that *sil* can coordinate a pathogenic program simultaneously across a genetically heterogeneous *S. pyogenes* population. QS in streptococci controls numerous pathogenic factors in a complex and sometimes overlapping network of regulatory pathways, suggesting that *fas* and *sil* could be fruitful targets for therapeutic intervention in GAS or GGS disease. But more research is required to fully understand how QS controls invasive infection in these species.

#### 2.2.3. *Lactobacillus* Systems

Unlike *agr* in *S. aureus*, control over toxin production and modulation of adhesion is not combined into a single regulon in *Lactobacillus* species. As mentioned above, *agr* signaling increases the production of adhesins in *L. plantarum*, but does not appear to regulate production of the antibacterial bacteriocin peptides. Instead, both of the *pln* and *plt* operons control the production of multiple bacteriocins [158]. Of particular note, both *plnC* and *plnD* are highly homologous to *agrA*, and *plnB* is closely related to *agrC* [68], although it is unclear how these various 2CRS components interact. *PlnA* and *pltA* are the propetides in their two systems, but they are unlike *agrD* in that their processed signal peptides are linear (Table 1) and are not readily soluble in aqueous solutions [68,180]. Furthermore, these two signal peptides share features with other bacteriocin peptides [181], suggesting that these quormones are bifunctional, serving both to signal lactobacilli and thwart the growth of neighboring species. Recent findings also suggest that *pln* peptides cooperatively regulate behaviors across *L. plantarum* strains, a behavior that counters the examplar *agr* alleles in *S. aureus* [182]. These discoveries highlight the need for care in development of broad-spectrum anti-QS compounds to avoid adversely affecting beneficial species like *Lactobacillus*.

## 3. Targeting *agr* and Analogues to Inhibit Disease

### 3.1. Overview

Pharmacologic interference with pro-virulence QS processes in pathogenic Firmicutes is an attractive strategy for restricting disease progression and potentially limiting the spread of infectious organisms. Targeting virulence factors, rather than bacterial growth, could retard pathology or even prevent infection without engendering resistance to chemotherapy that accompanies antibiotic therapy [14–16]. This strategy is complementary to the efforts of antibiotic stewardship programs that attempt to address skyrocketing antibiotic resistance at a time of limited antibiotic development [27,28]. Given that soil microbiota are a reservoir for resistance genes important in human pathogens [183] and that these resistance genes are present within the normal microbiota of the human gut [184] suggests that elimination of the genetic basis of resistance is not feasible and that new approaches are required to limit selective growth pressure that results in the proliferation of resistant organisms. In this regard, anti-virulence strategies are probably best employed as a component of an antibiotic stewardship program optimized for each pathogen that includes advanced infection control measures and appropriate antibiotic therapy. Such a strategy could extend the efficacy of existing and future antibiotics. Importantly, several of the species covered in this review with *agr* or *agr*-like quorum sensing systems and with known proclivities for developing antibiotic resistance, including *S. aureus*, *E. faecalis*, and *C. difficile*, represent some of the most common causes of health care associated infections that result in significant morbidity and mortality as well as increased health care costs. Therefore, anti-virulence strategies for these pathogens provided either by vaccination or by drug inhibition could have a significant impact on public health.

Currently, skin and skin structure infections comprise roughly 90% of all reported *S. aureus* infections [185], and from Section 1.4 it is clear that inhibiting *agr* function early in infection could prove highly effective in limiting disease development. Furthermore, survey evidence suggests that *S. aureus* persistently colonizes the mucus membranes and epithelium of 30% of healthy adults, making a blockade of QS-driven pathology and transmission to limit community-acquired disease an attractive prospect. Another potential disease where inhibition of *agr* could be beneficial is osteomyelitis arising from traumatic injury, where bone tissue is contaminated from direct contact with *S. aureus*-colonized epithelial and mucosal barriers. Prophylactic antibiotic therapy against MRSA is often prescribed for orthopedic surgery and in response to traumatic injury [186–188], and animal models have demonstrated the efficacy of this therapy in reducing experimental infection rates [189–191]. Replacing antibiotic prophylaxis with anti-QS treatments could serve as a way to inhibit the earliest stages of *S. aureus* infection and allow host innate immune defenses to respond to injury, without promoting antibiotic resistance. Combining approaches like enhanced infection control measures with anti-*agr* treatment could further reduce the spread of CA-MRSA by restricting the ability of *S. aureus* to infect new, healthy patients.

However, anti-*agr* treatments will not benefit all cases of *S. aureus* infection. While the majority of *S. aureus* infections are *agr*+, chronically infected patients, especially many with HA-MRSA, often carry mixed populations of *agr*^+^ and *agr*^−^*S. aureus* [5,6,102], and in these cases blocking *agr* function might limit severe disease pathology but it is unlikely to allow host defenses to completely resolve infection. Vancomycin insensitivity in clinical isolates is associated with the loss of *agr* function and expression, often through acquiring mutations that up-regulate repressors of *agr* such as σ^B^ [101,108,192], and so treating VISA infections to block *agr* is unlikely to be efficacious. This is also likely true of targeting *agr* function in *S. aureus* biofilm infections on medical implants and in ventilator associated pneumonias, as loss of *agr* function leads to thicker biofilms [93,106–108], although it could be a useful strategy to treat *agr*-dependent *S. epidermidis* biofilms [193].

**Figure 3.**
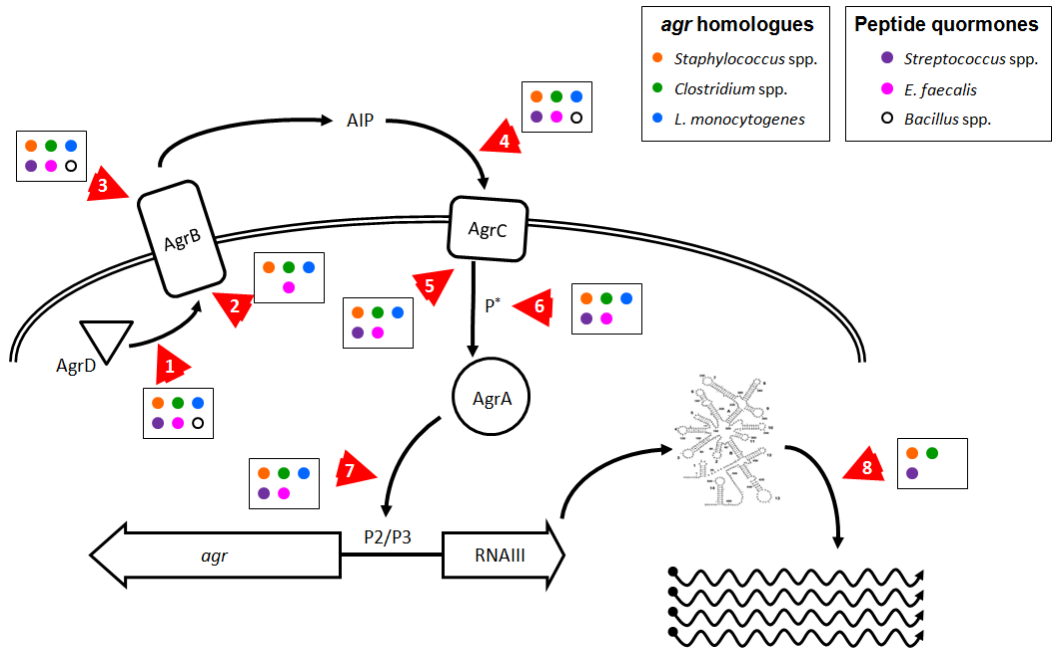
Known targets in *agr*-homologues and other peptide quormone QS systems with the potential for chemotherapeutic intervention to inhibit or retard virulence. Colored dots indicate which species are reported to possess *agr* homologues or analogues at the indicated step. (1) Binding and C′ terminal cleavage of AgrD/propeptide by AgrB/endopeptidase. (2) Cyclization of cleaved propeptide by AgrB. (3) Export of partially formed AIP/signal peptide by endopeptidase/export channel. (4) Binding of AIP by AgrC/cognate receptor module. (5) Dimerization and/or activation of the AgrC/receptor module HPK. (6) Phosphorylation and activation of AgrA/regulatory module by AgrC/receptor HPK. (7) Binding of consensus sequences by AgrA/regulatory module. (8) Binding of target mRNAs by RNAIII/*fsr*/VR-RNA and post-transcriptional regulation of gene expression.

As we have shown here, there are numerous pathogens where inhibition of *agr* or *agr*-like pathogenic QS systems could prove effective in controlling disease or reducing infection transmission. Reducing *agr*-regulated production of exotoxins in *L. monocytogenes* and in *Clostridium* species could improve the ability of the infected host to respond to infection and enhance bacterial clearance. Additionally, the use of probiotic strains that produce anti-*agr* compounds [130] could prevent *agr*-dependent disease due to these gut pathogens. Targeting *fas* or *sil* in *S. pyogenes*, *plc/pap* in *B. cereus*, and *fsr* in *E. faecalis* could reduce tissue invasion and apoptosis or death of host cells. Whether anti-QS treatments in these species would prove effective in limiting disease remains to be ascertained. Molecular targets involved in *agr* and *agr*-like pro-virulence QS systems in Firmicutes that could be affected by chemotherapeutic intervention are shown in Figure 3 and discussed further below.

### 3.2. Development of Synthetic Anti-agr Compounds

Blocking *agr* function to reduce the development of *S. aureus* pathologies *in vivo* has previously been established. Wright *et al.* demonstrated in the mouse dermonecrosis model that ulcers and abscesses caused by infection with an *agr* I strain could be blocked by administration of inhibitory concentrations of exogenous AIP II peptide [126]. Vaccination with hapten-linked AIP-4 allowed for the production of anti-AIP-4 sera providing passive immunity that reduced the pathology of an *agr* IV strain [117]. These proofs of concept clearly show that targeting *agr* to control virulence works. However, these therapies are severely restricted in that they are *agr* type specific, necessitating that *S. aureus* infections be *agr* typed before treatment, unless one were to administer multiple inhibitory peptides or pooled antisera active against all four *S. aureus agr* types. These limitations are inherent in any therapy that specifically targets AIP production or recognition due to the hypervariability inherent in the different *agr* operons. But, the functionality of AgrB, C and A is the same across all *agr* types, which holds true for how RNAIII regulates the virulon, and it is highly likely that compounds effective against these targets in multiple *S. aureus agr* types, and possibly even across different *agr*^+^ species, will be discovered.

Due to its high prevalence as both a nosocomial and community-acquired pathogen, *S. aureus* pathogenesis has been studied extensively and numerous experimental tools have been developed to examine *agr*-mediated QS, and these allow for relatively easy development of screens for anti-pathogenic anti-*agr* therapies. For example, a first-pass high-throughput screen could be developed using a *S. aureus* strain with a fluorescent reporter, such as the *S. aureus* Newman-GFP strain developed by Xiong *et al.*, where GFP expression is driven by the RNAIII promoter [194]. Promising compounds would specifically inhibit *agr*-driven fluorescence without bactericidal or bacteriostatic effects, and would be tested further to check they had no deleterious effect upon bacterial growth, membrane potential and membrane permeability. Follow-up experiments would include several *in vitro* assays to measure compound effects on specific *agr* regulon markers like α-hemolysin and capsule polysaccharides to confirm that the inhibitory molecule was not simply interfering with the fluorescence of the GFP reporter. Successful candidate drugs would at last be tested in *in vivo* models of *S. aureus* disease, such as the mouse dermonecrosis model, to investigate their bioavailability and pharmacokinetics. Then, thanks to reagents such as the suite of *agr*-GFP reporter strains generated in multiple *agr* types by Kavanaugh *et al.* [75], compound efficacy could be quickly assessed across the four *S. aureus agr* types.

The strategy outlined here for finding anti-QS effectors is one possible approach, appropriate for screening a library of small molecules, such as the NIH Molecular Library Screening Center. Computer-aided virtual screening of chemical libraries for QS inhibitors of the *lasR* sensor in *Pseudomonas aeruginosa* has identified several existing drugs with strong similarities to known anti-QS molecules [195], and a similar approach virtually identified novel compounds that partially inhibit the DNA-binding activity of *S. aureus* AgrA [196]. Use of these *in silico* methods may shorten the time required to select compounds to physically test against single targets with associated datasets, such as protein crystal structure, or the structure-activity relationships of known but clinically unfeasible inhibitors.

### 3.3. Inhibition of Pro-Virulence rRNAs

RNAIII may be the single most important target in the *S. aureus* virulence regulon, as it alters the expression of dozens of genes contributing to *S. aureus* pathogenesis [1] as well as many other genes controlling metabolism and biofilm production [1,2,83,96]. Reducing or inhibiting expression of RNAIII, or altering its ability to post-transcriptionally regulate virulence factor expression, should be the most efficacious way to restrict the pathogenesis program in *S. aureus* and possibly related species. Targeting this molecule could encompass both prophylactic and therapeutic applications. Surgical implants and grafts could be treated pre-surgery with an anti-RNAIII compound to reduce the dissemination of *S. aureus* from tenacious biofilms.

A direct approach would be to interfere with the action of RNAIII (Figure 3, arrow 8), which could be achieved in *S. aureus* by disrupting or destabilizing the secondary structure of the regulatory RNA molecule, decreasing the half-life of dsRNA species, or interfering with the mechanism of action of regulatory RNAs. Because there are multiple regulatory RNAs in *S. aureus* that exert control over a wide range of bacterial activities [83], broad interference with rRNA mechanisms could adversely affect bacterial survival. This is evident in the recent report by Olson *et al.*, where chemical inhibition of the RNAse RnaP decreased mRNA turnover, leading to reduced pathogenesis in a mouse model of *S. aureus* sepsis. However, this treatment was also bacteriostatic which could explain the observed reduction in disease [197]. If this approach could be fine-tuned to directly target the rRNAs responsible for virulence regulation, then this method could prove effective against other pathogenic *Staphylococcus* species, and possibly work to limit the effects of FasX in *S. pyogenes* and VR-RNA in *C. perfringens* [33,63,145,146].

In addition to directly targeting RNAIII to limit its ability to activate virulence factor expression in *S. aureus*, interfering with AgrA provides another opportunity to ameliorate disease development. Preventing the phosphorylation and activation of AgrA by AgrC (Figure 3, arrows 5 and 6), or blocking the ability of AgrA to bind DNA (Figure 3, arrow 7), would prevent RNAIII expression and block the RNAIII regulon and also abolish the smaller AgrA-driven virulence program. Leonard *et al.* recently published their efforts to identify molecular patterns which prevent AgrA from binding DNA at the P3 site, with moderate success *in vitro* [196], but it remains unclear whether their target, a hydrophobic cleft in the LytR domain, provides sufficient leverage to fully block AgrA function.

Another problem is the high degree of homology between AgrAC and other LytTRS family members, several of which are vital to *S. aureus* growth and survival. Siamycin I, a peptide effective against HIV fusion with human T cells, attenuates the ability of GBAP to activate FsrC and gelatinase production in *E. faecalis*, but its effects on other LytTR family members has not yet been ascertained [198,199]. Moreover, it is toxic for *Enterococcus* at doses sufficient for *fsr* inhibition. This example demonstrates that small molecule inhibitors must be exquisitely targeted to AgrA or AgrC alone. Small molecule inhibitors of the kinase domain of AgrC or of AgrA could also prove useful in limiting or blocking disease development in *L. monocytogenes*, Group A streptococci, and *E. faecalis*. In contrast, some RNPP family members do not employ a phosphorelay like AgrC/AgrA, and instead signal through other enzymes like the Rap phosphatases [159], requiring an entirely different class of compounds to inhibit.

### 3.4. Blocking the Production and Action of AIP

As mentioned in Section 1, production and recognition of the thiolactone-containing signaling molecule AIP is a highly sequence-specific process which can be inhibited at several points by AIP molecules from other *agr* types or by peptide analogs (Figure 3, arrows 1–5). Current technology facilitates the design and synthesis of numerous peptides or modified peptide analogs that can inhibit *agr*-mediated QS *in vitro*. Two studies clearly demonstrate effective inhibition of multiple *agr* alleles at nanomolar peptide concentrations using a single, common peptide analog to AIP molecules [81,200], but the efficacy of these peptides in preventing or reducing disease *in vivo* has not been demonstrated. Peptide-based cross-type inhibition of *agr* during skin infection *in vivo* has been shown in a proof-of-concept paper, with a single, high dose of AIP 2 transiently limiting *agr* activation but without an apparent effect on pathology [126]. Given the observation that the biological lifetime of an AIP molecule in solution *in vivo* is about 3 hours targeting *S. aureus* infections with an AIP-mimetic peptides as an anti-virulence therapy suffers from severe limitations, and by extension, the rational design of inhibitory molecules based off of the structure of AIP is of limited use. However, there are several unique aspects of the *agr* system which may be targeted effectively by non-peptide molecules.

AgrB is unusual in that it combines several disparate functions into a single enzyme (Figure 3, arrows 1–3). While peptide analogs could be generated to irreversibly antagonize the active site which binds, cleaves, and cyclizes AgrD, the enzyme structure could also be destabilized or forced into an inactive conformation by a small molecule, an approach for which there are *in vivo* examples from drug studies in mammals (for example FK506 Binding Proteins, the mTOR pathway, and PPARs [201–203]). Another approach would be identification of small molecule inhibitors that bind AgrB or an associated exporter to prevent export of AgrD. Because the mechanism by which AIP is transported from the cytoplasm is unknown, the feasibility of blockading AgrD export remains to be determined. There are numerous examples from the pharmacological literature demonstrating that export channels in both prokaryotes and eukaryotes can be targeted specifically to inhibit transporter functions. Ambuic acid, an antifungal agent, weakly and partially inhibits the production of AIP 1 in *S. aureus* and GBAP in *E. faecalis*, but the mechanism of action remains unclear [204]. Either approach could serve as a prophylactic treatment for *S. aureus* infection and to prevent biofilm maturation and dissemination. But without identification of high affinity irreversible inhibitors such therapies could prove ineffective for extant infections in which *agr*-mediated QS is ongoing and producing ever more AgrB, AgrD and AIP. Additionally, peptide analogs or compounds working in these ways would need to function at the extracellular face of AgrB or first gain entry to the bacterial cell before they could block its cytoplasmic functions. However, this type of treatment should provide compounds effective against all of the species discussed in this review and represents a strategy likely to have significant commonality.

AIP itself can be targeted, as demonstrated by the numerous examples listed in Section 1.5 detailing how the innate immune system interferes with *agr*-mediated QS, and also by the anti-AIP-4 vaccine [117]. How current pharmacological approaches with small molecules could improve upon the existing, natural defenses against AIP and *S. aureus* QS is difficult to predict. However, interfering with AgrC in its ability to bind AIP and dimerize (Figure 3, arrows 4 and 5) could prove a fruitful target. Several reports detail attempts to generate synthetic peptide analogs capable of inhibiting all types of AIP simultaneously, but many of these compounds suffer from a poor affinity to at least one of the *agr* alleles, and these analogs remain untested in *in vivo* models of *S. aureus* disease [81,126,200,205]. But as with AgrB, small molecules that alter AgrC's conformation so that it fails to bind AIP or fails to dimerize are possible to discover through bulk screens. However, the prospect of developing compounds acting against peptide quormone production or recognition are no better in bacteria with only a single QS allele: a peptide-derived analog of the *fsr* quormone in *E. faecalis* never completely inhibits gelatinase production [206]. As this approach targets the ability of *S. aureus* to recognize and respond to AIP, this treatment should prove effective both as prophylaxis and *post facto* treatment for *Staphylococcus* pathogens, as well as members of the *B. cereus* group, *L. monocytogenes*, groups A, B, and G streptococci, and against *E. faecalis*, all of which employ signaling peptides or possess known AgrC homologues. The difficulty in targeting AgrC is similar to finding molecules that inhibit AgrA, as there are numerous 2CRS and HPK homologues to AgrC, so any anti-*agr* compounds that work against AgrC would need to be tested extensively to confirm they do not demonstrate broader antimicrobial effects.

## 4. Conclusions

Targeting non-vital, pro-pathogenic QS mechanisms in *S. aureus* and G- pathogens like *E. coli* and *P. aeruginosa* has been suggested as a way to curtail bacterial virulence without engendering resistance [14,15]. One of the complicating factors in targeting the AHL and AI-2 pathways in G- pathogens, as well as some G+ species, is the large and diverse array of organic small molecules and their cognate intracellular sensors employed, making it difficult to predict whether a treatment that works against these QS mechanisms in any given pathogen could work against any other. However, the marked degree of homology and distinct functional features shared between *agr* family members and orthologues across the Firmicutes offers the prospect of developing a small suite of therapies that can inhibit pathogenesis in numerous community-acquired and nosocomial pathogens beyond *S. aureus*.

## Figures and Tables

**Table 1.** *agr* homologues, analogues, and peptide pheromone systems in G+ pathogens, their effects on virulence, and their signal peptide sequences. Cysteines and serines in color highlight the residues required for thiolactone and lactone ring formation. Gray and white banding is to highlight individual bacteria.

**Species**	***agr*Homologues/Analogues**	**Discovery Method**	**Up-Regulated Behavior**	**Signal Peptide Name and Sequence**^1^		
***Staphylococcus aureus***	*agr* (*agrBDCA)*	Mutants [2,30]	Virulence (toxins, capsule) [1,31,32]	Sa AIP 1	YST 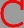 DFIM
				Sa AIP 2	GVNA 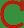 SSLF
				Sa AIP 3	IN 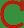 DFLL
				Sa AIP 4	YST 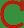 YFIM
***S. epidermidis***	*agr*	Mutants, genomics [33–35]	Virulence (toxins), biofilm [34,36–38]	AIP-Se 1	DSV 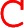 ASYF
				AIP-Se 2	*YNP 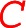 NSYL*
				AIP-Se 3	*YNP 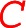 SAYL*
***S. lugdunensis***	*agr*	Genomics [20,39]		HKU09-01	DI 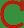 NAYF
				N920217	DMN 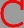 NGYF
***S. saprophyticus***	*agr*	Genomics [40]		*agr*	*TINP 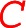 FGYT*
***S. pseudintermedius***	*agr*	Genomics, proteomics [41,42]		Si AIP 1	RIPT 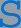 TGFF
				*agr* II	*RIPI 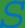 TGFF*
				*agr* III	*KIPT 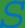 TGFF*
				*agr* IV	*KYPT 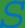 TGFF*
***Staphylococcus* spp.**	*agr*	Mutants, genomics, proteomics [18,37,43]	Virulence (hemolysins, surface proteins) [20,37,40,43]		
***Clostridium botulinum***	*agrD1*, *agrD2*	Mutants [44]	Sporulation (*agrD1*), virulence (toxins) (*agrD2*) [44]	*agrD1*	*ACYW 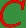 VYQP*
				*agrD2*	*ADSA 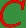 HLGI*
***C. difficile***	*agr*, *agrD2*	Genomics [44,45]		*agrD*	*ANST 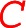 PWII*
				*agr2*	*NSA 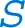 SWVA*
***C. perfringens***	*agrBD*	Mutants, genomics [18,21,46, 47]	Virulence (toxins, proteases), sporulation [21,46,47]		*ATSA 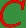 IWFT*
***Enterococcus faecalis***	*fsr* (*fsrBDCA*)	Mutants, genomics [18,48–50]	Virulence (proteases), biofilms [49,50]	GBAP	QN 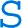 PNIFGQWM
***Listeria monocytogenes***	*agr*	Mutants, genomics [18,51,52]	Biofilm, virulence (internalization, toxins) [51–53]	4b *agr*	*MSKA 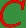 FMFV*
				DG119D	*RLAS 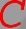 LYTQ*
				*agr*	
***Lactobacillus plantarum***	*lam* (*lamBDCA*)	Mutants [54]	Adhesion [54]	LamD558	LVMC 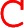 VGIW

	**Peptide Quormone Analogues**				
***Bacillus cereus***	*pap, plc*	Mutants, genomics [55,56]	Sporulation, virulence (cytotoxicity, toxins), stress responses [55–58]	PapR I	ADLPFEF
				PapR II	SDMPFEF
				PapR III	NEVPFEF
				PapR IV	SDLPFEH
				PapRa	*CSIPYEY*
***B. thuringiensis***	*pap*	Mutants [59]	Virulence (toxins) [59,60]		
***Bacillus* spp.**	*plc, pap*	Mutants, genomics [18,55,61]	Sporulation, virulence (toxins) [55,56]		
***Streptococcus pyogenes***	*comC, comRS, fas*, *sil*	Mutants, genomics [62–64]	Virulence (invasion, toxins), competence, biofilms [62–66]	CSP	MRLSKFFRDFILQRKK
				XIP	*SAVDWWRL*
				SilCR	DIFKLVIDHISMKARKK
***Streptococcus* spp.**	*comC, comRS, sil*	Mutants, genomics [62,64,67]	Competence, biofilms [64,67]	XIP	*GLDWWSL*
***Lactobacillus* spp.**	*pln, plt*	Mutants, genomics [18,54]	Adhesion, bacteriocin production [54,68]	PlnA	KSSAYSLQMGATAIKQVKKLFKKWGW
				PltA	EQLSFTSIGILQLLTIGTRSCWFFYCRY

1 Sequences in italics denote peptide sequences identified through genomics methods but not yet confirmed through mass spectrometry or other chemical methods.
